# Supplementary material for: The Impact of the 2019 European Guideline for Cardiovascular Risk Management: A Cross-Sectional Study in General Practice
Source: J Clin Med. 2020 Jul 7;9(7):2140. doi: 10.3390/jcm9072140 (PMC7408902; doi:10.3390/jcm9072140)
Supplement: Supplementary file 1 [file jcm-09-02140-s001.pdf]

Supplementary Materials: The Impact of the 2019 European Guideline for Cardiovascular Risk Management: A Cross-Sectional Study in General Practice

Rahel Meier, Yael Rachamin, Thomas Rosemann and Stefan Markun

1. Implementation of ESC-Risk Classification in the FIRE Database

1.1. Implementation of ESC Risk Categories

In both guidelines, cardiovascular (CV) risk categories (low, moderate, high and very high) are defined by either a systematic coronary risk estimation (SCORE) [1] or the presence of morbidities and risk factors. We used a multistep identification process to determine the according CV risk category at every time point for each patient with available data in the FIRE database.

1.1.1. SCORE Based Risk Identification

The calculation of the SCORE value based on systolic blood pressure, total cholesterol, age, gender and smoking status. For the implementation of the "Systematic COronary Risk Estimation" (SCORE), some assumptions and categorizations were made: cholesterol values and systolic blood pressure values were valid for a maximum of 5 years. A SCORE was only assigned if a systolic blood pressure value and a cholesterol value were concurrently available. If the values were updated, the SCORE was reassigned. We adapted categories for age, cholesterol and systolic blood pressure proposed by the European Society of Cardiology (ESC) guidelines as illustrated in supplementary Table S1. Based on these categorized values, we assigned the according SCORE of both guidelines 2016 and 2019 [2,3]. The difference between the SCORE 2016 and 2019 is depicted in supplementary Table S2. Based on the values of the SCORE patients are assigned to the different CV risk categories: low- risk: SCORE < 1, moderate-risk: SCORE ≥1 and <5, high-risk: SCORE ≥5 and <10 and very-high risk: SCORE ≥10.

Table S1. Scale transitions defined for parametric values.

Variables	ESC Category	Classification for SCORE 2016	Classification for SCORE 2019
pat_age	70		68–70
	65	63–67	63–67
	60	58–62	58–62
	55	53–57	53–57
	50	45–52	45–52
	40	40–44	40–44
Cholesterol (mmol/L)	4	≤4.49	≤4.49
	5	4.5–5.49	4.5–5.49
	6	5.5–6.49	5.5–6.49
	7	6.5–7.99	6.5–7.99
sBP (mmHg)	180	≥170	≥170
	160	150–169	150–169
	140	130–149	130–149
	120	≤129	≤129

Table S2. Comparison between SCORE 2016 and SCORE 2019, red: SCORE 2019 is higher, green: SCORE 2016 is higher.

Age Group	syst. BP	Ratio 2019/2016															
		women								men							
		non-smokers				smokers				non-smokers				smokers			
age 65	180	1.0	0.8	0.8	0.8	0.8	0.8	0.7	0.8	1.0	1.0	1.0	1.0	0.8	0.8	0.8	0.8
	160	1.0	1.0	1.0	1.0	0.8	1.0	0.9	0.9	1.2	1.2	1.1	1.1	0.9	0.9	0.9	0.9
	140	1.0	1.5	1.5	1.0	1.0	1.0	1.0	0.8	1.3	1.3	1.2	1.2	1.0	1.0	1.0	1.0
	120	2.0	2.0	1.0	1.0	1.0	1.0	1.0	1.0	1.5	1.3	1.7	1.3	1.0	1.2	1.2	1.0
		4	5	6	7	4	5	6	7	4	5	6	7	4	5	6	7
		cholesterol								cholesterol							
		women								men							
		non-smokers				smokers				non-smokers				smokers			
age 60	180	0.7	1.0	1.0	0.8	0.8	1.0	0.8	0.9	1.0	1.0	1.0	1.0	0.8	0.9	0.8	0.9
	160	1.0	1.0	1.0	1.0	1.0	0.8	1.0	0.8	1.3	1.0	1.0	1.0	0.9	0.9	0.9	0.8
	140	1.0	1.0	1.0	1.0	1.0	1.0	1.0	1.0	1.5	1.0	1.0	1.0	0.8	1.0	1.0	1.0
	120	1.0	1.0	1.0	1.0	2.0	1.0	1.0	1.0	1.0	1.0	1.0	1.0	1.0	1.0	1.0	1.0
		4	5	6	7	4	5	6	7	4	5	6	7	4	5	6	7
		cholesterol								cholesterol							
		women								men							
		non-smokers				smokers				non-smokers				smokers			
age 55	180	1.0	1.0	1.0	1.0	1.0	1.0	1.0	1.0	1.0	1.0	1.0	1.0	1.0	1.0	1.0	0.9
	160	1.0	1.0	1.0	1.0	1.0	1.0	1.0	1.0	1.0	1.0	1.0	1.0	1.0	0.8	0.8	0.9
	140	1.0	1.0	1.0	1.0	1.0	1.0	1.0	1.0	1.0	1.0	1.0	1.0	1.0	1.0	0.8	0.8
	120	1.0	1.0	0.0	1.0	1.0	1.0	1.0	1.0	1.0	1.0	1.0	1.0	1.0	1.0	0.7	1.0
		4	5	6	7	4	5	6	7	4	5	6	7	4	5	6	7
		cholesterol								cholesterol							
		women								men							
		non-smokers				smokers				non-smokers				smokers			
age 50	180	1.0	1.0	1.0	1.0	2.0	2.0	1.0	1.5	1.0	1.0	1.0	1.0	1.0	1.3	1.0	1.0
	160	1.0	1.0	1.0	1.0	1.0	1.0	1.0	2.0	1.0	1.0	1.0	1.0	1.0	1.0	1.0	1.0
	140	1.0	1.0	1.0	1.0	1.0	1.0	1.0	1.0	1.0	1.0	1.0	1.0	0.5	1.0	1.0	1.0
	120	1.0	1.0	1.0	1.0	1.0	1.0	1.0	1.0	0.0	1.0	1.0	1.0	1.0	1.0	0.5	1.0
		4	5	6	7	4	5	6	7	4	5	6	7	4	5	6	7
		cholesterol								cholesterol							
		women								men							
		non-smokers				smokers				non-smokers				smokers			
age 40	180	1.0	1.0	1.0	1.0	1.0	1.0	1.0	1.0	1.0	1.0	1.0	1.0	2.0	2.0	3.0	1.5
	160	1.0	1.0	1.0	1.0	1.0	1.0	1.0	1.0	1.0	1.0	1.0	1.0	1.0	1.0	1.0	2.0
	140	1.0	1.0	1.0	1.0	1.0	1.0	1.0	1.0	1.0	1.0	1.0	1.0	1.0	1.0	1.0	2.0
	120	1.0	1.0	1.0	1.0	1.0	1.0	1.0	1.0	1.0	1.0	1.0	1.0	1.0	1.0	1.0	1.0
		4	5	6	7	4	5	6	7	4	5	6	7	4	5	6	7
		cholesterol								cholesterol							

1.1.2. Morbidity Based Risk Identification

We identified morbidities and risk factors specified in the ESC guidelines (CV disease, diabetes mellitus (DM) with target organ damage, DM with major risk factors (age, smoking status, dyslipidaemia, hypertension and obesity) [4], severe/moderate chronic kidney disease (CKD), markedly elevated single risk factors, DM without risk factors/target damage) based on International classification of primary care (ICPC-2)codes [5], anatomical therapeutic chemical (ATC) [6], codes of

morbidity-specific medication, laboratory values or vital signs. Duration of morbidity was indefinite for all except markedly elevated single risk factors for which we defined a maximal validity of 5 years. As specified in the ESC-Guidelines of 2016 and 2019, the according risk categories were assigned to the patients. Whenever multiple identifications were present risk, estimations were valid until the patient was assigned to a higher risk category. We did not allow for a downscaling of risk. All morbidities, risk factors, the differences between the two guidelines and the according identification scheme is depicted in supplementary Table S3.

Table S3. Cardiovascular risk categories based on morbidities and risk factors with the identification scheme used in the database; entries marked in red could not be identified within the FIRE database.

According to ESC 2016			According to ESC 2019			Identification scheme
**CV risk category**	**Disease classification**	**Disease sub-classification**	**CV risk category**	**Disease classification**	**Disease sub-classification**	
very high risk	CVD	Other documented CVD	very high risk	ASCVD	Other documented ASCVD	ICPC = K74 or K76
		Myocardial infarction			Myocardial infarction	ICPC = K75
		Acute coronary syndrome			Acute coronary syndrome	
		Coronary revascularisation			Coronary revascularisation	ATC code regexp B01AC04|B01AC22|B01AC25|B01AC24
		Coronary bypass graft surgery			Coronary bypass graft surgery	
		other artery revascularisation procedures			Other artery revascularisation procedures	
		Stroke			Stroke	ICPC = K90 OR K91
		Transient ischemic attack			Transient ischemic attack	ICPC = K89
		Peripheral arterial disease			Peripheral arterial disease	ICPC = K92
	DM with target organ damage	Microalbuminuria		DM with target organ damage	Microalbuminuria	Albumin/creatinin quotient > 30
		Retinopathy			Retinopathy	ICPC = F83
		Neuropathy			Neuropathy	ICPC = N94
	DM with a major risk factor	Age (men ≥55 years; women ≥65 years)		DM with at least three major risk factors	Age (men ≥55 years; women ≥65 years)	men ≥55 years; women ≥65 years
		Smoking			Smoking	ICPC = P17

		Dyslipidaemia			Dyslipidaemia	Triglyceride >1.7 mmol/L<br>or Total Cholesterol >5 mmol/L<br>or LDL-Cholesterol > 3 mmol/L<br>or (sex = female and HDL-Cholesterol ≤1.2 mmol/L)<br>or (sex = male and HDL-Cholesterol ≤1 mmol/L)
		Hypertension			Hypertension	ICPC = K85 or K86 or K87<br>or two BP measurements > 140/90 mmHg<br>OR ATC regexp C02/C03A/C03EA01/C0[78]/C09[AB]
		Fasting plasma glucose 5.6–6.9 mmol/L			Fasting plasma glucose 5.6–6.9 mmol/L	
		Abnormal glucose tolerance test			Abnormal glucose tolerance test	
		Obesity			Obesity	ICPC = T82 or BMI > 30
		Abdominal Obesity			Abdominal Obesity	
		Family history of premature CVD			Family history of premature CVD	
				Family history of CVD with another major risk factor		
	Severe CKD	GFR < 30		Severe CKD	GFR < 30	GFR_CKDEPI < 30
high risk	single risk factors	Cholesterol > 8	high risk	single risk factors	Cholesterol > 8	Cholesterol > 8 mmol/L
					LDL > 4.9	LDL > 4.9 mmol/L
		BP > 180/110			BP > 180/110	BP > 180/110 mmHg
	DM without risk			DM without risk		ICPC = T89 or T90<br>or HbA1c > 6.5%<br>or ATC regexp A10

	factors/target damage			factors/target damage		
	moderate CKD	GFR 30–59		moderate CKD	GFR 30–59	GFR_CKDEPI < 60 & GFR_CKDEPI ≥ 30
moderate risk			moderate risk	young DM	T1DM age < 35, T2DM age < 50 with DM duration < 10 y.	

CVD: Cardiovascular disease; ASCVD: Atherosclerotic cardiovascular disease; DM: Diabetes mellitus; T1DM: Diabetes mellitus type 1; T2DM Diabetes mellitus type 2; LDL: Low-density lipoprotein; HDL: High density lipoprotein GFR: Glomerular filtration rate; GFR_CKDEPI: Glomerular filtration rate calculated according to Chronic Kidney Disease Epidemiology Collaboration; BP: blood pressure; ICPC: International classification of primary care; ATC: Anatomical Therapeutic Chemical;.

1.1.3. Combination of Risk Estimations

We combined the two identification schemes to a single risk classification system. Whenever multiple reasons for classification were available at the same time point, the one with the higher risk level was adopted. After the first initial risk classification was set, an update was only considered when an increase in risk occurred. The only exception was in patients whose risk category based on an "untreated" SCORE, for which we allowed for a downscaling of risk category.

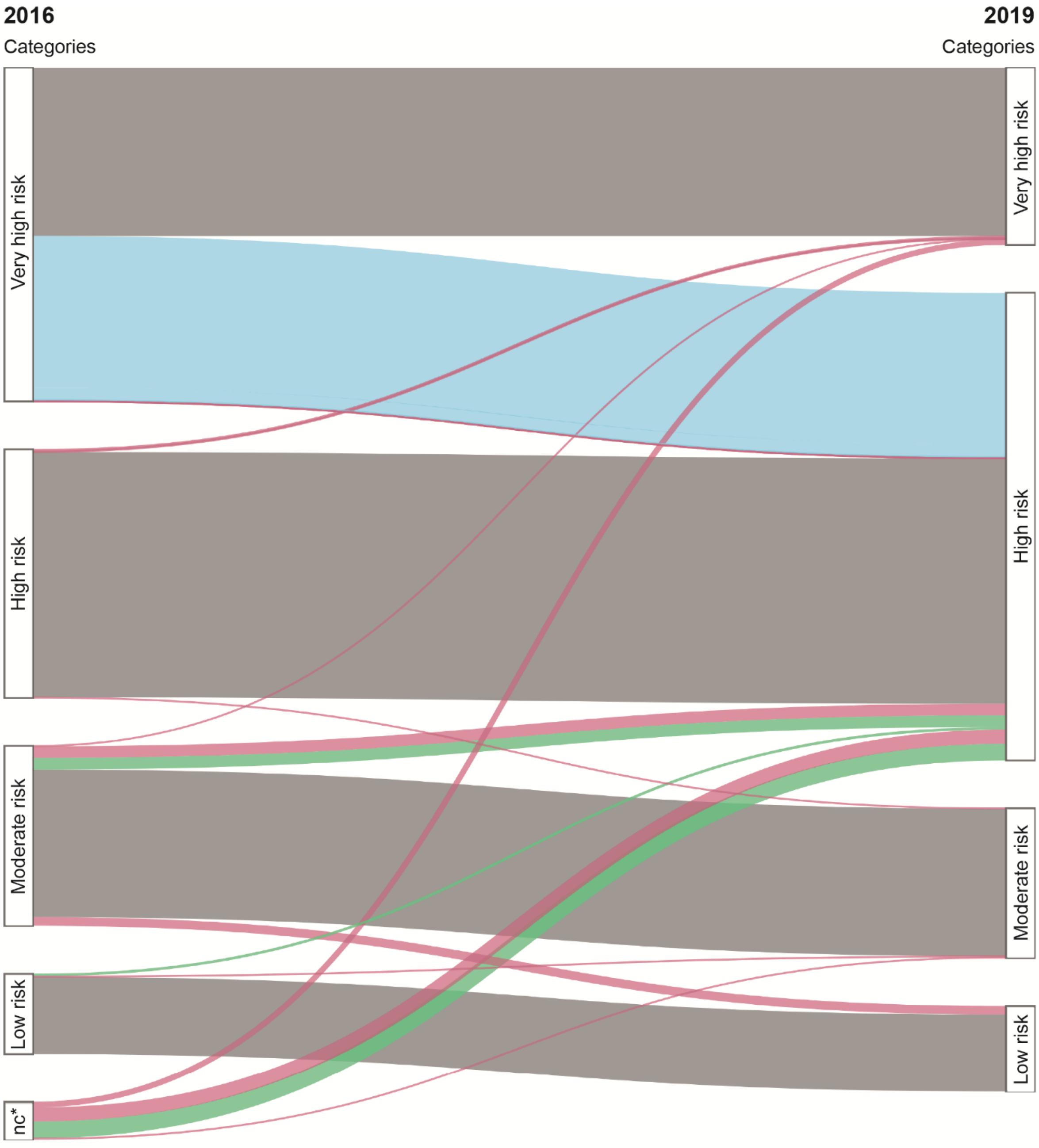

** = no classification*

Figure S1. Impact of 2019 guideline on risk classification. Reasons for reclassifications are indicated by color: blue—adaptation in the identification scheme for diabetes with major risk factors, pink—SCORE adaptation, green—adaptation in single elevated risk factor identification.

References

1. Conroy, R.M.; Pyorala, K.; Fitzgerald, A.P.; Sans, S.; Menotti, A.; De Backer, G.; De Bacquer, D.; Ducimetière, P.; Jousilahti, P.; Keil, U. et al. Estimation of ten-year risk of fatal cardiovascular disease in Europe: the SCORE project. *European Heart Journal* **2003**;*24(11):*987-1003. doi: 10.1016/s0195-668x(03)00114-3
2. Mach, F.; Baigent, C.; Catapano, A.L.; Koskinas, K.C.; Casula, M.; Badimon, L.; Chapman, M.J.; De Backer, G.G.; Delgado, V.; Ference, B.A. et al. 2019 ESC/EAS Guidelines for the management of dyslipidaemias: lipid modification to reduce cardiovascular risk: The Task Force for the management of dyslipidaemias of the European Society of Cardiology (ESC) and European Atherosclerosis Society (EAS) *European Heart Journal* **2020,** *41,* 111–188. doi: 10.1093/eurheartj/ehz455
3. Catapano, A.L.; Graham, I.; De Backer G.; Wiklund, O.; Chapman, M.J.; Drexel, H.; Hoes, A.W.; Jennings, C.S.; Landmesser, U.; Pedersen , T.R. et al. 2016 ESC/EAS Guidelines for the Management of Dyslipidaemias. *European Heart Journal* **2016**, *37(39):*2999-3058. doi: 10.1093/eurheartj/ehw272
4. Cosentino, F.; Grant, P.J.; Aboyans, V.; Bailey, C.J.; Ceriello, A.; Delgado, V.; Federici, M.; Filippatos, G.; Grobbee, D.E.; Hansen, T.B. et al. 2019 ESC Guidelines on diabetes, pre-diabetes, and cardiovascular diseases developed in collaboration with the EASD: The Task Force for diabetes, pre-diabetes, and cardiovascular diseases of the European Society of Cardiology (ESC) and the European Association for the Study of Diabetes (EASD). *European Heart Journal* **2020**, *41,* 255–323 doi: 10.1093/eurheartj/ehz486
5. International Classification of Primary Care, Second edition (ICPC-2). Available online: https://www.who.int/classifications/icd/adaptations/icpc2/en/ (accessed on 26 May 2020)
6. WHO Collaborating Centre for Drug Statistics Methodology. ATC-Structure and principles Oslo, Available online: https://www.whocc.no/atc/structure_and_principles/ (accessed 09.07.2019)
